# Bacteriophages and Their Role in Food Safety

**DOI:** 10.1155/2012/863945

**Published:** 2012-12-18

**Authors:** Sanna M. Sillankorva, Hugo Oliveira, Joana Azeredo

**Affiliations:** Institute for Biotechnology and Bioengineering (IBB), Centre for Biological Engineering, University of Minho, Campus de Gualtar, 4710-057 Braga, Portugal

## Abstract

The interest for natural antimicrobial compounds has increased due to alterations in consumer positions towards the use of chemical preservatives in foodstuff and food processing surfaces. Bacteriophages fit in the class of natural antimicrobial and their effectiveness in controlling bacterial pathogens in agro-food industry has led to the development of different phage products already approved by USFDA and USDA. The majority of these products are to be used in farm animals or animal products such as carcasses, meats and also in agricultural and horticultural products. Treatment with specific phages in the food industry can prevent the decay of products and the spread of bacterial diseases and ultimately promote safe environments in animal and plant food production, processing, and handling. This is an overview of recent work carried out with phages as tools to promote food safety, starting with a general introduction describing the prevalence of foodborne pathogens and bacteriophages and a more detailed discussion on the use of phage therapy to prevent and treat experimentally induced infections of animals against the most common foodborne pathogens, the use of phages as biocontrol agents in foods, and also their use as biosanitizers of food contact surfaces.

## 1. Introduction

Everyday people worldwide buy and consume a diversity of products of animal and plant origin expecting these products to be safe. However, annually, millions of people become ill, are hospitalized, and die due to a variety of foodborne pathogens transmitted through foods. For instance, the World Health Organization estimated in their fact sheet of March 2007 (no. 237) that in 2005, over 1.8 million people died due to diarrhoeal diseases. Furthermore, annually in the USA alone, there are roughly 48 million illnesses, 128,000 hospitalizations, and even 3,000 deaths caused by foodborne pathogens [1]. 

Regardless of modern technologies, good manufacturing practices, quality control and hygiene, changes in animal husbandry, agronomic process, and in food or agricultural technology, food safety is continuously challenged by changes in lifestyle and consumer demands (e.g., ready-to-eat products) and also by the increase of international trade [2]. The most efficient means for limiting the growth of microbes are good production hygiene, a rational running of the process line, and a well-designed use of biocides and disinfectants [3]. However, even when acceptable cleaning procedures are applied, bacteria are found in foods and food contact surfaces [4]. Food products may become contaminated at different stages along the food chain, from growth or production until the final consumption. Furthermore, the inherent ability of pathogens to attach to living and inert surfaces, where they start living in microbial communities known as biofilms, and become highly tolerant to varied antimicrobial agents [5] also contributes to the pathogen prevalence in foods and food contact surfaces. So, to meet the primary goal of any food safety program, the consumer protection, new food preservation techniques have to be continually developed to meet current demands, in order to control the emerging pathogens and their impact at global scale.

## 2. Bacteriophage: Charting the Path to Food Safety

 (Bacterio)phage, viruses specifically infecting bacteria, are harmless to humans, animals, and plants. Since the discovery of phages in 1915, they have been extensively used not only in human and veterinary medicine but also in various agricultural settings. Being obligatory parasites, upon multiplication by taking over host protein machinery, phages can either cause cell lysis to release the newly formed virus particles (lytic pathway) or lead to integration of the genetic information into the bacterial chromosome without cell death (lysogenic pathway).

Towards a food safety perspective, strictly lytic phages are possibly one of the most harmless antibacterial approaches available. 

**Figure 1:**
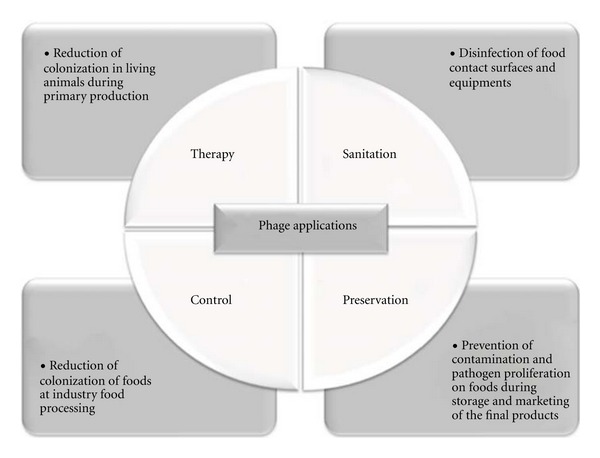
Feasible applications of phages along the food chain towards an increased food safety (adapted from Greer [18]).

Phages offer advantages as biocontrol agents for several reasons: (i) high specificity to target their host determined by bacterial cell wall receptors, leaving untouched the remaining microbiota, a property that favors phages over other antimicrobials that can cause microbiota collateral damage; (ii) self-replication and self-limiting, meaning that low or single dosages will multiply as long as there is still a host threshold present, multiplying their overall antimicrobial impact; (iii) as bacteria develop phage defense mechanisms for their survival, phages continuously adapt to these altered host systems; (iv) low inherent toxicity, since they consist mostly of nucleic acids and proteins; (v) phages are relatively cheap and easy to isolate and propagate but may become time consuming when considering the development of a highly virulent, broad-spectrum, and nontransducing phage; (vi) they can generally withstand food processing environmental stresses (including food physiochemical conditions); (vii) they have proved to have prolonged shelf life. Phages are readily abundant in foods and have been isolated from a wide variety of raw products (e.g., beef, chicken) [6, 7], processed food (e.g., pies, biscuit dough, and roast turkey) [8], fermented products (e.g., cheese, yoghurt) [9], and seafood (e.g., mussels and oysters) [8, 10]. This suggests that phages can be found in the same environments where their bacterial host(s) inhabit, or once were present and that phages are daily consumed by humans. Furthermore, the use of antibiotics prophylactically and therapeutically in farm animals has become a major concern due to their possibility of contributing to the declining efficacy of the antibiotics used to treat bacterial infections in humans and leading to the alarming emergence of superbugs like *Salmonella* DT104 and the methicillin-resistant and multidrug-resistant *Staphylococcus aureus*. The use of phages to promote food safety can be basically done at four different stages along the food chain (Figure 1). 

Reduction of pathogens colonization in animals during primary production (*phage therapy*) is a strategy followed in primary production just before slaughter or during animal growth to reduce the probability of cross-contamination with the animal feces during food processing. For example, it is estimated that a reduction of 2 log on the *Campylobacter* loads in poultry intestines is sufficient to diminish 30-fold the incidence of campylobacteriosis associated with consumption of chicken meals [11]. The proof of principle of phage therapy in animals was already established for several pathogens (detailed description below). Phages can be administered orally, incorporated in drinking water or food, to control *Salmonella* and *Campylobacter* in poultry, or by spray to target avian pathogenic *E. coli* in poultry, and orally/rectally to control *E. coli* in ruminants.

Reduction of colonization on foods (*biocontrol*) during industrial food processing can be accomplished by applying phages directly on food surfaces, for example, in case of meats, fresh produce, and processed foods or even mixed onto raw milk. Experimental data reveals that phages are very effective against actively growing bacteria and lose effectiveness in nongrowing bacteria [12]. In these cases, effective control could be achieved, applying high titres of phages to control pathogens by “lysis from without” mechanisms [6, 13] or whenever phages start to replicate immediately after the food begins to warm (i.e., during preparation, handling, and/or consumption).

In food industry, biofilms are found on the surfaces of equipment used, for example, in food handling, storage, or processing, especially in sites that are not easy to clean or to sanitize. Some of the work using phages against *in vitro* biofilms formed by spoilage and pathogenic bacteria show that under ideal conditions significant viable cell reductions are achieved and thus, their use for *biosanitation* is promising although very challenging due to the diversity of bacteria found in different settings.

Phages are also excellent as food *biopreservation* agents since they are reported to lyse hosts at temperatures as low as 1°C [14, 15], limiting growth of pathogenic and spoilage bacteria on refrigerated foods (specially psychrotrophic bacteria); once the foods are taken to room temperature, phages can further control their proliferation [16].

## 3. Phages Targeting Different Food Pathogens

The global incidence of foodborne disease and costs associated are difficult to estimate, however cost at least $7 billion dollars each year in medical expenses and lost productivity in the United States according to the United States Department of Agriculture's Economic Research Service [17]. The actual figure is higher since this estimate reflects illnesses caused only by the major foodborne pathogens. The most frequently reported pathogens from animal origin, responsible for such impact, are separately discussed in this section, focusing on their contamination sources and their harms for animals and humans and furthermore summarizing the recent phage interventions and main outcomes. We also briefly address some of the common phytopathogens and discuss phage applications carried out in an attempt to decrease crop diseases and product loss.

## 4. Foodborne Pathogens from Animal Origin

The four main foodborne pathogens from animal origin are accounted to be *E. coli*, *Campylobacter*, *Salmonella,* and *Listeria*. These bacteria are all common contaminants of ruminants, poultry, and swine and are usually carried in their gastrointestinal tract asymptomatically. Research on the use of phages against foodborne pathogens from animal origin has mainly focused on the optimization of preharvest interventions where the phage administration routes and delivery processes have received most attention and also on the optimization of postharvest strategies. The usage of phages as a preharvest strategy is made directly by administering phages to livestock to prevent animal illness and/or also to minimize the pathogen carriage in the gastro-intestinal tract, thereby preventing pathogen entry to the food supply. Postharvest strategies are based on the use of phages directly on animal carcasses in an attempt to sanitize the products.

*Escherichia coli* is a gram-negative bacterium. Serotype O157 : H7 in particular, classified as Shiga toxin-producing *E. coli*, is a well-known food poisoning pathogen. Its major reservoir comprises ruminants and, as it can survive well under intestinal conditions, if proper care is not taken during slaughter, the contents of the intestines, fecal material, or dust on the hide may contaminate meats [19]. The most common route of *E. coli* transmission to humans is via undercooked contaminated food, while water and raw milk are assumed to be related to cross-contamination events, by direct or indirect contact with feces. This microorganism is highly virulent and a public health threat because ingestion of a concentration as low as 10 cells is able to cause infection [1, 20, 21].

Recent pre- and postharvest phage research targeting *E. coli* is listed in Table 1.

Recent phage therapy to decrease *E. coli* levels on farm animals has focused mainly on poultry and ruminants. 

Application of phages to poultry has been successful to prevent fatal respiratory infections in broiler chickens [22–24]. Several different approaches have been used; however, aerosol spraying and intramuscular (i.m.) injection have given the best results and reduced significantly the mortality of broiler chicken. Despite these results, phage administration via addition to bird drinking water proved to be inefficient in protecting the birds from fatal *E. coli* respiratory infections. 

Although some successful results of phage therapy in ruminants have been reported using oral delivery of phages via direct administration or addition to drinking water and/or feed, the majority of the recently published papers suggest that oral treatment is unsuccessful in reducing *E. coli* levels (see Table 1). 

The main speculated causes for the failure of oral treatments have been reported to be (i) nonspecific binding of phages to food particles and other debris in the rumen and gastrointestinal tract [25]; (ii) phage inactivation upon contact with the acidic conditions of the abomasum [26]; (iii) causing an insufficient number of orally phages reaching the gastrointestinal tract [27]. An interesting approach to reduce coliphage inactivation has been described by Stanford and colleagues in 2010. These authors successfully encapsulated phages in polymeric matrices which resisted *in vitro* acidic conditions and furthermore, once delivered orally to steers caused reduction of *E. coli* levels [28].

Essentially, oral delivery of phages has reported to be successful either using a cocktail of phages or combined with rectal treatment. For instance, a combined oral/rectal treatment using phages KH1, SH1 was able to reduce *E. coli* levels compared to oral treatment alone or to individual phage administration, but still this combined oral/rectal treatment using a phage cocktail did not cause total eradication of the pathogen from cattle [32]. Also, a cocktail of phages CEV1 and CEV2 were reported to lead to more than 99.9% of reduction of *E. coli* in sheep guts [36]. However, although reductions of *E. coli* levels in different organs have been described, phages still fail in reducing fecal shedding [29, 35], and only in one published work the authors managed to reduce fecal shedding duration by 14 days [28]. In 2008, Niu and colleagues investigated two different sampling techniques: fecal grab and rectoanal mucosal swab for surveillance of *E. coli* O157 : H7 [42] and to study the role of phage as a mitigation strategy. Their study showed discrepancy between fecal and rectoanal mucosal swab sampling in 63 of the 213 positive samples from experimentally inoculated feedlot steer. This shows that sampling procedures can significantly influence the merit of phage and that prudence must be taken to validate phage therapy for *E. coli* O157 : H7 control [42].

All postharvest interventions reported since 2000 have been effective in reducing *E. coli* levels from fresh produce and meats. Also, phage application to *E. coli* contaminated food contact surfaces has led to significant reductions proving that phage can be used safely for equipment and food contact surface sanitation.

The successful use of coliphages has led to the development of a phage-product (EcoShield (Intralytix)) which received regulatory approval from the Food and Drug Administration (FDA) in 2011. This product can be used on red meat parts and trims intended to be ground (Food Contact Notification no. 1018) and has proved to eliminate from 95 to 100% of *E. coli* O157 : H7. Finalyse (Elanco Food Solutions) is another product on the market using naturally occurring phages specific for *E. coli* O157 : H7. Finalyse is sprayed on cattle to reduce the load of *E. coli*, prior to its entering the beef packing facility (preharvest strategy).

*Campylobacter* is a genus of gram-negative, spiral, motile, and microaerophilic bacteria with an optimal growth temperature around 41°C. *C. jejuni* and *C. coli* members are considered to be major aetiological agents of enteric diseases worldwide. *Campylobacter* is the most commonly reported zoonosis in Europe (EFSA 2011), and *C. jejuni*, in particular, is estimated to cause approximately 845,000 illnesses, 8,400 hospitalizations, and 76 deaths each year in the USA [1]. This widespread infection is explained because ingestion of low doses (400–500 cells) [43] can cause campylobacteriosis typically characterized by fever, bloody diarrhea, and acute abdominal pain [44]. *Campylobacter* is capable of colonizing the intestine of poultry and cattle, and thus infection is mostly acquired by fecal-oral contact, ingestion of contaminated foods (i.e., raw meat and milk contaminated through feces), and waterborne through contaminated drinking water [45–47]. The widespread disease and economic impact on agriculture and food industries has led to the development of various approaches to contain this infection using bacteriophages (Table 2).

Phage preharvest interventions reported so far have been successful in reducing *Campylobacter* numbers in the cecal content and feces of experimentally infected broilers and have not caused any adverse health effects. However, some degree of resistant phenotypes, recovered from phage-treated chickens, has been reported [48–50], of which some were found to display clear evidence of genomic rearrangements [51]. Scott and colleagues demonstrated that *Campylobacter* virulent phages have the potential to activate dormant prophages, leading to rapid pathogen evolution towards the development of phage-resistant phenotypes. These authors also showed that whilst pathogen evolution can be rapid, resistance to the therapeutic bacteriophage is associated with a decreased fitness to environment, specifically meaning that phage-resistant phenotypes exhibited a decreased ability to colonize the gastrointestinal tract [51]. These genomic instability findings suggest that *C. jejuni* adopt this strategy to temporarily survive local environmental pressures, including phage predation and competition for resources. The key elements for the success of phage therapy against *Campylobacter* in broiler chickens are a proper selection of phage, the dose of phage applied, and the time elapsed after administration [48].

As for postharvest strategies, two studies have been reported (see Table 2) in which phages reduced *Campylobacter* contamination following a “lysis from without” mechanism. This suggests that *Campylobacter* phages can be used as a tool for biocontrol purposes.

It is known that *Campylobacter* attaches and forms biofilms on surfaces as a measure to overcome environmental stresses, such as aerobic conditions, desiccation, heating, disinfectants, and acidic conditions frequently encountered in food environments [52]; however, to date, we were only able to find one report evaluating the efficacy of *Campylobacter* phages of disrupting biofilm formed on glass. In this study, phages were able to reduce by 1 to 3 log the viable cell counts under microaerobic conditions; however, after treatment above 84% of the surviving bacteria were resistant to the two phages applied [53].

*Salmonella,* is a genus of gram-negative facultative intracellular species, is considered to be one of the principal causes of zoonotic diseases reported worldwide. *Salmonella* serovars can colonize and persist within the gastrointestinal tract, and so human salmonellosis is commonly associated with consumption of contaminated foods of animal origin. *Salmonella* infections cost nearly 3 million euros in EU per annum in health care systems [17, 54]. *Salmonella enterica* serovars, Enteritidis and Typhimurium, are responsible for the majority of *Salmonella* outbreaks, and most events are reported to be due to consumption of contaminated eggs and poultry, pig, and bovine meats, respectively [55]. *Salmonella* is also a known spoilage bacterium in processed foods. Once ingested, this microorganism can cause fever, diarrhea, abdominal cramps, and even life-threatening infections [56]. To prevent such infections, a number of studies on animal phage therapy have been reported where phages were used to prevent or reduce colonization and diseases in livestock. All recent studies are summarized in Table 3.

Phage therapy of experimentally *Salmonella*-infected poultry and swine animals has been successful and significantly decreased *Salmonella* in major tissues such as ileum and cecal tonsils. Although these results have been mostly obtained within contrived laboratory conditions, the success of postslaughter phage employment, to lower the risk of cross-contamination, will only be determined after extending these studies to poultry farms.

Apart from one report in 2001, where the multivalent Felix01 phage was used, all other *in vivo* experiments performed recently were carried out using cocktails of two to six phages. Furthermore, Ma and colleagues (2008) have encapsulated phage Felix01 in a chitosan-coated Ca-alginate spheres [57] and have found in *in vitro* studies that this technique preserves phage viability upon exposure to acidic conditions; however, to our knowledge, these encapsulated phages have not yet been tested *in vivo*. Valuable outputs have been made in the last couple of years in terms of using phages for *Salmonella* biocontrol. Today, two phage-products are available: (1) BacWash from OmniLytics Inc. which received, in 2007, USDA's Food Safety and Inspection Services approval to be commercialized and applied as a mist, spray, or wash on live animals prior to slaughter; (2) BIOTECTOR S1 phage product from CheilJedang Corporation that is to be applied on animal feed to control *Salmonella* in poultry.

Unlike phage preharvest strategies on animals, several postharvest strategies have adopted the use of only one phage and not a cocktail. All *Salmonella* phages reported have been able to decrease the number of viable cells present on raw meats, processed and ready-to-eat foods, and fresh produce. Furthermore, the combined treatment of phage and *Enterobacter asburiae*, a strain exhibiting antagonistic activity against *Salmonella*, to control this pathogen on tomatoes, mung bean sprouts, and alfalfa seeds, represents a highly promising, chemical-free approach. However, in some settings phages were found to be readily immobilized by the food matrix and, although retaining infectivity, they lost the ability to diffuse and infect target cells [59].

*Listeria monocytogenes* is a gram-positive, motile, and facultative intracellular bacterium, that can grow under several food matrices and storing conditions (e.g., high salt levels, low pH, lack of oxygen, and low temperatures) [60]. Invasive infection by *L. monocytogenes *causes listeriosis and is transmitted to humans with 10^3^ CFU/mL levels. It is often associated with contaminated minimally processed food such as ready-to-eat (RTE) products, poultry, and dairy products or related to cross-contamination after the heat treatment process of foods stored at low temperatures [61, 62]. Despite its low incidence, estimated to be in order of 2 of 10 reported cases per million per year in Europe [63], its pathogenicity causes a high mortality rate of approximately 255 deaths each year in the USA alone according to the Centers for Disease Control and Prevention (CDC) [1]. As so, contingency measures have led to the establishment of a limit for common RTE foods of 100 CFU per gram in EU and a zero tolerance policy in the USA.

Considering the sources of *Listeria* outbreaks, phage research has focused on postharvest applications (Table 4).

The first two studies in Table 4 were carried out using a combination of phage and a nisin, a broad spectrum antibacteria peptide used during production to extend shelf life by suppressing gram-positive spoilage and pathogenic bacteria. While in ground beef the phage-nisin combination revealed to be ineffective, this strategy had a synergistic effect once added to melon and apple resulting in an improved reduction of *Listeria* compared to phage or nisin alone. The efficacy of phage-nisin mixture was however significantly reduced in apples on the account of a decline of phage numbers possibly due to the low pH. Phage biocontrol should therefore be optimized separately for each food matrices under study. Four other studies have used phage P100, which was highly effective in inhibiting *Listeria* growth at storage temperatures for several days (see Table 4). Like in *Campylobacter*, only one article, by Soni and Nannapaneni (2010), on the efficacy of phages against biofilms was found for *Listeria monocytogenes*. These authors evaluated the ability of P100 against 21 *L. monocytogenes* strains belonging to 13 serotypes and found that P100 reduced by 3.5 to 5.4 log/cm^2^ the viable cells present in stainless steel surfaces [64]. Nonetheless, studies carried out on a variety of experimentally contaminated meats, fresh produce, and processed food among others show that biocontrol is influenced by phage contact time and phage dose, regardless of higher or lower temperature. Despite this fact, the results conducted on RTE foods and meats using LMP-102 phage preparation (six-phage cocktail) allowed the commercialization of ListShield phage product from Intralytix. Also, LISTEX P100 phage-based-product (from EBI Food Safety) is being commercialized with a GRAS status (generally recognized as safe) to prevent *Listeria* contamination on food products and food processing facilities. 

*Staphylococcus aureus* is a gram-positive bacterium, is considered to be a major threat to food safety [1, 90], and also is the most common agent of mastitis in dairy cows [91]. CDC estimates that staphylococcal food poisoning, which results from the consumption of foods containing sufficient amounts of one or more preformed enterotoxins [91, 92], is 242.148 cases annually in the USA [1]. The mechanisms described for the contamination of foods with *S. aureus* can be of animal or human origin, such as due to the infection and colonization of livestock or farm workers and even due to the human handling of the food products [93]. *S. aureus* can cause toxin-mediated diseases within 1 to 6 h after consumption of contaminated foods; nevertheless the symptoms are usually mild and most people recover within 1–3 days [94]. The annual estimated loss worldwide due to mastitis in adult dairy cows is of 35 billion US dollars [95]. Phage research has focused on the treatment of mastitis in lactating dairy cows and mostly in dairy food products (Table 5).

The experimental work of phage therapy on dairy cattle to control *S. aureus* on teats shows that, although there was no increase in somatic cell counts in milk samples indicating that the phages did not irritate the animal [96, 97], there were no statistically different outcomes between phage-treated and placebo groups [97]. This suggests that the use of phage as teat washes or sanitizers will not reduce the incidence of *S. aureus* mostly due to the barriers to phage-mediated bacteria lysis which are present in the bovine mammary gland. The suggested reasons for the inefficacy of phage K are the degradation or inactivation of the phage particles within the gland and the inhibitory effects of raw milk [97]. Also, it has been reported that milk whey prevents phages from reaching their host cell surface [98] due to the agglutination of *S. aureus *cells upon contact with raw whey [98, 99]. However, the work by García et al. [100] on the use of phages in curd manufacturing processes provides evidence that phages are stable and active during enzymatic curd formation suggesting that pH is, in fact, the most crucial inactivation factor. Furthermore, these authors postulated that the activity in milk of the phages used in their work was due to their dairy origin. 

Postharvest applications in pasteurized milk show that the use of combined phage treatments with nisin and high hydrostatic pressure could synergistically be used to reduce staphylococcal contamination compared to each treatment alone [101, 102]. Nevertheless, care should be taken in regard to combined phage-nisin application since nisin-adapted strains can seriously compromise phage activity [101]. Inactivation of *S. aureus* has also been accomplished in both fresh and hard-type cheese using a phage cocktail during cheese manufacturing [103]. While for fresh cheese staphylococcal cells could not be detectable until the end of the curdling process (after 24 h), in hard cheeses, the presence of staphylococcal strains continuously dropped to 1.24 log CFU per gram at the end of ripening [103] providing evidence that phages can be successfully used to control *S. aureus *in dairy food products.

Today, there are phage-based diagnostic tools available to detect *S. aureus*, including antibiotic resistant (MRSA) and susceptible (MSSA) strains (Microphage, Inc.). 

Besides the five foodborne pathogens described above, several other foodborne pathogens are responsible for illnesses, hospitalizations, and deaths, such as *Clostridium* spp., *Shigella* spp., and *Vibrio* spp. Nonetheless, these are not discussed further as to date there is a limited number of articles on the use of phage against these pathogens.

## 5. Concluding Remarks and Future Perspectives

Soon after the discovery of phages, several studies produced negative results frequently associated with their inappropriate usage, including administration to treat viral and unknown agent diseases. Now, when once more the potential of phages is being evaluated mostly due to the increasing emergence of multiresistant strains, it is important not to repeat the same mistakes. Furthermore, the adoption of phage administration and delivery routes, control procedures, concentrations, and timings of application among other parameters based on results found in the literature must be done with caution as the efficacy of each phage or cocktail of phages is highly dependent on the phage-host systems in study. Furthermore, sampling of experimentally contaminated animals and foods and their treatment/control with phage should mimic as much as possible the conditions found at preharvest and postharvest (slaughterhouse, processing, and at retail) stages. For example, to estimate the input of the slaughterhouse into the process chain, samples should be collected before and after cooling. Ready-to-eat food usually undergoes several processing stages, and thus it may be reasonable to verify the efficacy of phage after the process where phages were applied or, at least, at the end of the processing stage. Nevertheless, it is well evident that even after phage treatment, changes in food items still occur during transport and storage. Future studies should also focus on improving the general understanding of the mechanisms of phage resistance acquired by the hosts and the rate of elimination from the animal body. In addition, further evaluation of phage for biosanitation purposes is still not well documented for the pathogens listed above.

Although the results of the above-described studies appear to be encouraging, they should be interpreted cautiously. For instance, some phage studies have proven that phages are inefficient in reducing their host, such as phage delivery on foods and drinking water for controlling *E. coli* 0157 : H7 in poultry and ruminants, and reduced efficacy of some of the phages reported. Also, the emergence of phage-resistant phenotypes is reported by several authors; however this has not significantly affected the results of the phage trials on animals and can be managed with using phages which target those resistant phenotypes. Nonetheless, summarizing briefly the results described recently: (i) phage therapy is able to reduce foodborne pathogen levels in animals and consequently control the pathogen load on entry at the slaughterhouses; (ii) the strategies applied for phage biocontrol of pathogens in foods reduce significantly the levels in a variety of products and seem to be a promising alternative to traditional food safety and preservation measures; (iii) phage use in agricultural settings is as efficient or more than the conventionally used agents to control the growth of plant-based bacterial pathogens. However, the future of phages in food safety is further dependent on the regulatory agencies that still display uneasiness with using phages, mostly due to a scarcity of strong scientific evidence generated through fully controlled clinical trials under the supervision of ethical committees and in compliance with the highest regulatory standards of leading Western jurisdictions [90]. Also, there is a need to educate farmers, producers, and the general public about the advantages of their use. 

## Figures and Tables

**Table 1:** Pre- and postharvest *E. coli* O157 : H7 phage applications.

Year	Animal/product	Phage(s)	Strategy	Main outcome	Refs
Preharvest application					

2002	Poultry (broiler chicken)	SPR02	Air sac or drinking water	Air sac inoculation prevented mortality. Drinking water offered no protection	[24]
2002	Poultry (broiler chicken)	SPR02 and DAF6	Sprayed	Significant decrease of mortality but not complete protection	[22]
2003	Poultry (broiler chicken)	SPR02 and DAF6	Sprayed and i.m. injection	Aerosol spray effective only when applied immediately after bird challenge with *E. coli.* A single i.m. injection reduced mortality when applied immediately and 24 and 48 h after challenge	[23]
2003	Ruminant (lamb)	DC22	Oral delivery	No reduction of fecal shedding over 30 days	[29]
2006	Poultry (broiler chicken)	SPR02 and DAF6	i.m. injection into the left thigh	Only high phage titers (10^8^) reduced mortality	[30]
2006	Ruminant (sheep)	CEV1	Oral delivery	2 log CFU reduction within 2 days	[31]
2006	Ruminant (cattle)	Phage cocktail (KH1, SH1)	Oral/rectal delivery (via drinking water)	No reduction of CFU when applied orally. Combined oral/rectal treatment reduced CFU but did not eradicate it	[32]
2009	Ruminant (steer)	Phage cocktail	Oral/rectal delivery	Small fecal shedding reduction of oral/rectal compared to the rectal treatment and control	[33]
2010	Ruminant (steer)	Phage cocktail (wV8, rV5, wV7, wV11)	Oral delivery (gelatin capsules and in feed)	No reduction of fecal shedding of nalidixic acid-resistant *E. coli* O157 : H7, but duration of shedding was reduced by 14 days	[28]
2010	Poultry	Phage cocktail	Oral delivery and spray	Significant reduction of mortality in large scale animal experiments	[34]
2010	Ruminant (cattle)	Phage cocktail (e11/2, e4/1c)	Oral delivery	Rapid CFU decrease within 24 to 48 h, but no decrease in fecal shedding levels	[35]
2011	Ruminant (sheep)	Phage cocktail (CEV1, CEV2)	Oral delivery	Cocktail eradicated (>99.9%) the pathogen and is more effective than CEV1 alone	[36]

Postharvest application					

2004	Meat	e11/2, e4/1c, pp01	Applied on top	Eradication in seven of nine samples	[37]
2008	Fresh produce (tomato, spinach) and meat	Phage cocktail (ECP-100)	Applied on top/sprayed	94% and 100% reductions in CFU after 120 h and 24 h in tomato and spinach; 95% reduction in ground meat after 24 h at 10°C	[38]
2009	Fresh produce (lettuce, cantaloupe)	Phage cocktail (ECP-100)	Sprayed	Significant CFU reductions after 2 days at 4°C	[39]
2011	Fresh produce (lettuce, spinach)	Phage cocktail (BEC8)	Added to foods together with trans-cinnamaldehyde (TC)	No survivors detectable after 10 min of phage combined with the TC treatment	[40]
2011	Food surfaces (spinach blades)	Phage cocktail	Sprayed	4.5 log reduction CFU after 2 h of phage	[41]
2011	Food surfaces (steel, ceramic chips)	Phage cocktail (BEC8)	Applied on top	Eradication after 10 min at 37°C and after 1 h at 23°C	[40]

**Table 2:** Pre- and postharvest *Campylobacter* phage applications.

Year	Animal/product	Phage(s)	Strategy	Main outcome	Refs
Preharvest application					

2005	Poultry (broiler chickens)	ΦCP8, ΦCP34	Oral delivery in antacid suspension	Decrease of CFU between 0.5 and 5 log CFU/g in the cecal content over a 5-day period posttreatment	[48]
2005	Poultry (broiler chickens)	69, 71	Oral delivery	Reduction of CFU by 1 log within 5-day period post-treatment. Phage preventive treatment caused a delay in a colonization	[58]
2009	Poultry (broiler chickens)	CP220	Oral delivery	Reduction of 2 log CFU per g in cecal content after 48 h inoculated *C. jejuni* and *C. coli* birds with a single dose (7 log PFU) of CP220	[49]
2010	Poultry (broiler chickens)	Phage cocktail	Oral delivery (oral gavage and in feed)	Reduction levels of *C. coli* and *C. jejuni* in feces by 2 log CFU per g when administered by oral gavage and in feed	[50]

Postharvest application					

2003	Meat (chicken skin)	Φ2	Applied on top	1 and 2 log CFU reduction at 4°C using 10^7^ PFU per mL. 10^5^ and 10^3^ PFU per mL failed to decrease CFU	[58]
2003	Meat (chicken skin)	Φ29C	Applied on top	MOI 1 caused less than 1 log reduction in CFU; MOI 100–1,000 caused 2 log reductions in CFU	[49]
2008	Meat (raw and cooked beef)	Cj6	Applied on top	The largest reductions were recorded at high host cell density on both raw and cooked beef over a period of 8 days incubation at 51°C	[16]

**Table 3:** Pre- and postharvest *Salmonella* phage applications.

Year	Animal/product	Phage(s)	Strategy	Main outcome	Refs
Preharvest application					

2001	Poultry (chicken)	Phage cocktail	Oral delivery (direct and via feed)	Reduction of CFU in cecal counts between 0.3 and 1.3 log compared to controls birds	[65]
2001	Swine (pig)	Felix01	Oral delivery and i.m.	Reduction of CFU in the tonsils and cecum	[66]
2005	Poultry (broiler chickens)	CNPSA1, CNPSA3, CNPSA4	Oral delivery	Reduction of CFU by 3.5 orders of magnitude after five days	[67]
2005	Poultry (chickens)	Phage cocktail (Sa2, S9, S11)	Oral delivery phage/competitive exclusion	Reduction of CFU in cecum and ileum after phage cocktail and/or competitive exclusion treatment	[68]
2007	Poultry (broiler chickens)	Φ151, Φ25, Φ10	Oral delivery (antacid suspension)	Reduction of 4.2 log and 2.19 log with phages Φ151 and Φ25 within 24 h compared with control	[69]
2007	Poultry (broiler chickens)	Phage cocktail (CB4*ϕ*, WT45*ϕ*)	Oral delivery	Reduction of CFU in cecal tonsils after 24 h. No significant differences at 48 h compered to controls	[70]
2008	Poultry (chickens)	Phage cocktail	Oral delivery (coarse spray/drinking water)	Reduction of intestinal colonization of ten-day-old experimentally contaminated birds	[71]
2010	Swine (pig)	Phage cocktail		Reduction of colonization by 99.0 to 99.9% in the tonsils, ileum, and cecum	[72]
2011	Swine (weaned pigs)	Phage cocktail	Oral delivery	Significant reduction of CFU in the rectum	[73]
2011	Poultry (chickens)		Oral delivery (via feed)	Phage prevented horizontal transmission on six-week-old infected chickens	[74]

Postharvest application					

2001	Processed food (ripened cheese)	SJ2	Added to milk	No survival during 89 days in pasteurized cheeses containing phages (MOI 10^4^)	[75]
2001	Fresh produce (fresh-cut melon and apple)	Phage cocktail	Added to foods	Significant CFU reduction on melon but not on apple	[76]
2003	Meat (chicken skin)	P22, 29C	Applied on top	MOI 1 caused less than 1 log reduction in CFU; MOI 100-1,000 caused 2 log reductions in CFU and eradicated resistant strains	[13]
2003	Meat (chicken frankfurters)	Felix O1		Approx. 2 log reduction with a MOI of 1.9 × 10^4^	[21]
2004	Fresh produce (sprouting seeds)	A, B	Applied by immersion	Phage-A reduced CFU by 1.37 logs on mustard seeds. Cocktail resulted in a 1.5-log reduction in CFU in the soaking water of broccoli seeds	[77]
2005	Meat (broiler, turkey)	PHL 4	Sprayed	Phage treatments reduced frequency of *Salmonella* recovery as compared with controls	[78]
2008	Meat (raw/cooked beef)	P7	Applied on top	Reduction in CFU of 2-3 log at 5°C and approx. 6 log at 24°C	[16]
2009	Fresh produce (tomatoes)	Phage cocktail	Phage + *E. asburiae* JX1 added to food	Prevalence reduction of internalized *S. Javiana*, although the major suppressing effect was via antagonistic activity of *E. asburiae* JX1	[79]
2010	Fresh produce (mung bean sprouts and alfalfa seeds)	Phage cocktail	Phage + *E. asburiae* JX1 added to foods	Combined biocontrol with *E. asburiae* and phage suppressed pathogen growth on mung beans and alfalfa seeds	[80]
2011	Meat (pig skin)	Phage cocktail (PC1)	Applied on top	Above 99% reduction in CFU for MOI of 10 or above at 4°C for 96 h	[59]
2012	Ready-to-eat foods and chocolate milk	FO1-E2	Added to foods and mixed in milk	At 8°C no viable cells. At 15°C reduction of CFU by 5 logs on turkey deli meats and in chocolate milk and by 3 logs on hot dogs	

**Table 4:** Postharvest *Listeria* phage applications.

Year	Product	Phage(s)	Strategy	Main outcome	Refs
2002	Meat (ground beef)	Phage-nisin mixture	Applied on top	Phage-nisin mixture was effective in broth but not in buffer or on raw beef	[81]
2003	Fresh produce (melons, apples)	Phage cocktail (LM-103, LM-102) combined with nisin	Applied on top or sprayed	Phage caused a CFU reduction of 2.0 to 4.6 log in melons and only 0.4 log in apples. Phage + nisin reduced CFU by 5.7 (melon) and 2.3 (apple) log	[82]
2004	Fresh produce (honeydew melon)	Phage cocktail	Sprayed	Spraying melon pieces 0 h up to 1 h after *Listeria* challenge reduced CFU by 6.8 log units after 7 days of storage	[83]
2005	Processed food (red-smear soft cheese)	P100	Applied to surfaces during the rind washings	Reduction of CFU or complete eradication during the rind washings	[84]
2009	Processed food (cooked ham)	P100	Applied on top	Rapid 1 log reduction of CFU. 2 log reduction after 14 to 28 days of storage	[85]
2009	Fresh produce (ready-to-eat products)	A511, P100	Added to foods	In liquid foods, eradication of bacterial cells. On solid foods reduction of CFU by up to 5 log	[86]
2010	Meat (salmon fillet)	P100	Applied on top	Complete inhibition of growth at 4°C for 12 days, at 10°C for 8 days, and at 30°C for 4 days	[64]
2010	Meat (catfish fillets)	P100	Applied on top	Reduction of CFU by 1.4–2.0 log units at 4°C, 1.7–2.1 logs at 10°C, and 1.6–2.3 logs at 22°C	[87]
2011	Processed food (red-smear cheese)	A511	Applied on top	CFU counts dropped 3 logs after 22 days. Repeated application of A511 further delayed re-growth	[88]
2011	Processed food (ready-to-eat chicken)	FWLLM1	Added to foods	Reduction of CFU by 2.5 log at 30°C. At 5°C, regrowth was prevented over 21 days	[89]

**Table 5:** Preharvest and Postharvest *Staphylococcus aureus* phage applications.

Year	Animal/product	Phage(s)	Strategy	Main outcome	Refs
Preharvest application					

2005	Ruminant (dairy cattle)	K, CS1, DW2	Syringe applied into the teat sinus	No detectable increase in somatic cell counts in milk	[96]
2006	Ruminant (lactating dairy cattle)	K	Intramammary infusions	Cure rate comparable in phage-treated and saline-treated quarters. No large increase of somatic cell count in the milk when phage was infused into quarters with* S. aureus* infection	[97]

Postharvest application					

2005	Raw and ready-to-eat foods (milk products and derivatives)	K	Added to milk	Phages adsorption was reduced in raw milk and replication inhibited	[99]
2006	Raw food (raw milk whey)	K	Added to raw milk whey	Phage attachment and lysis inhibition due to adsorption of whey proteins to the *S. aureus* cell.	[98]
2007	Processes food (milk curd)	Cocktail (Φ88 and Φ35)	Added to pasteurized whole milk	*S. aureus *not detectable after 4 h at 25°C in acid curd and total clearance within 1 h of incubation at 30°C in renneted curd. The addition of the phage cocktail to milk prior to acid and enzymatic curd manufacture eliminates *S. aureus* by up to 6 log units	[100]
2008	Ready-to-eat foods (pasteurized milk)	Cocktail (Φ35, Φ88) with nisin	Applied to foods and mixed in milk	Nisin-phages application decreased *S. aureus* by 1 log unit more than in each antimicrobial agent alone (24 h at 37°C). Nisin-resistant phenotypes acquired resistance to phage, but phage resistance did not necessarily confer nisin resistance	[101]
2012	Ready-to-eat foods (pasteurized milk)	Cocktail (philPLA35, philPLA88 with high hydrostatic pressure (HHP)	Applied to foods and added to milk	Combination of HHP and phage resulted in *S. aureus* elimination within the 48 h regardless of the initial contamination level (1 × 10^6^ or 1 × 10^4^ CFU per mL)	[102]
2012	Ready-to-eat foods (cheese)	vB_SauS-phi-IPLA35, vB_SauS-phi-SauS-IPLA88	Added to pasteurized milk vat	Phage cocktail led to undetectable limits of *S. aureus *after 6 h in fresh cheese and continuous reductions in hard cheese. In curd a reduction of 4.64 log CFU per g was obtained compared with control	[103]
